# Genome-Wide DNA Methylation Profiling of Peripheral Blood Mononuclear Cells Reveals Epigenetic Signatures in Autism Spectrum Disorder

**DOI:** 10.3390/ijms27104161

**Published:** 2026-05-07

**Authors:** Thanit Saeliw, Wasana Yuwattana, Chayanit Poolcharoen, Marlieke Lisanne van Erp, Songphon Kanlayaprasit, Natchaya Vanwong, Valerie W. Hu, Pon Trairatvorakul, Weerasak Chonchaiya, Tewarit Sarachana

**Affiliations:** 1 Chulalongkorn Autism Research and Innovation Center of Excellence (ChulaACE), Department of Clinical Chemistry, Faculty of Allied Health Sciences, Chulalongkorn University, Bangkok 10330, Thailand; thanit.sa@chula.ac.th (T.S.); natchaya.v@chula.ac.th (N.V.); 2 The Ph.D. Program in Clinical Biochemistry and Molecular Medicine, Department of Clinical Chemistry, Faculty of Allied Health Sciences, Chulalongkorn University, Bangkok 10330, Thailand; 6271009037@student.chula.ac.th (W.Y.); 6473001537@student.chula.ac.th (M.L.v.E.); 3 The M.Sc. Program in Clinical Biochemistry and Molecular Medicine, Department of Clinical Chemistry, Faculty of Allied Health Sciences, Chulalongkorn University, Bangkok 10330, Thailand; 6470002637@student.chula.ac.th; 4 Center of Excellence for Medical Genomics, Department of Pediatrics, Faculty of Medicine, Chulalongkorn University, Bangkok 10330, Thailand; songphon.k@chula.ac.th; 5 Excellence Center for Genomics and Precision Medicine, King Chulalongkorn Memorial Hospital, The Thai Red Cross Society, Bangkok 10330, Thailand; 6 Department of Biochemistry and Molecular Medicine, School of Medicine and Health Sciences, The George Washington University, Washington, DC 20037, USA; valhu@gwu.edu; 7 Center of Excellence for Maximizing Children’s Developmental Potential, Division of Growth and Development, Department of Pediatrics, Faculty of Medicine, Chulalongkorn University, Bangkok 10330, Thailand; pon.t@chula.md (P.T.); weerasak.ch@chula.ac.th (W.C.)

**Keywords:** autism spectrum disorder, epigenetics, DNA methylation, differentially methylated positions, differentially methylated regions, heterogeneity

## Abstract

Autism spectrum disorder (ASD) is a complex neurodevelopmental disorder caused by the interaction between genetic and environmental influences, potentially mediated by epigenetic mechanisms such as DNA methylation. Genome-wide DNA methylation profiling was performed using the Infinium MethylationEPIC v2.0 array on peripheral blood mononuclear cells (PBMCs) from 100 children with ASD and 50 typically developing controls. Differential methylation analyses were conducted by adjusting for age, sex, and estimated blood-cell-type composition as covariates. Functional enrichment, SFARI gene-overlap analysis, and cross-cohort validation were performed. We identified 3507 differentially methylated positions (DMPs) in the ASD cohort. Functional enrichment revealed pathways involved in neuronal signaling, synaptic activity, and immune regulation, suggesting coordinated neurodevelopmental and immune processes in ASD. Stratification by clinical severity demonstrated common and unique biological characteristics between the moderate and severe ASD groups. Furthermore, DMP-associated genes significantly overlapped with high-confidence ASD risk genes from the SFARI database and established transcriptomic signatures of neurodevelopmental disorders. Comparisons with independent post mortem brain tissue and peripheral blood datasets revealed partial overlap and directional concordance. However, the strength of concordance varied across datasets and was limited in the most directly comparable peripheral blood cohort. Our findings suggested that DNA methylation profiling of PBMCs provided peripheral epigenetic signatures and candidate loci for further validation in larger independent cohorts.

## 1. Introduction

Autism spectrum disorder (ASD) is a complex neurodevelopmental disorder characterized by impairments in social communication and restricted interests and repetitive behaviors, which exhibit clinical heterogeneity [1]. ASD arises from complex interactions between genetic predisposition and environmental influences, potentially mediated by epigenetic mechanisms such as DNA methylation [2,3,4]. There is accumulating evidence that environmental factors, such as exposure to endocrine-disrupting compounds (e.g., bisphenol A, phthalates, and heavy metals), contribute to the risk of ASD [5,6,7]. These environmental influences may interact with genetic susceptibility through various molecular mechanisms, leading to neurodevelopmental alterations. At the molecular level, multiple layers of dysregulation have been reported in ASD, including gene expression [8,9], non-coding RNA [10,11], alternative splicing [12,13,14], and widespread changes in DNA methylation patterns [15,16,17].

The DNA methylation mechanism is increasingly recognized as a contributor to the etiology of ASD [18,19]. This mechanism involves the addition of a methyl group to cytosine residues at CpG sites, influencing gene expression by modulating chromatin structure and transcription factor accessibility. Several studies have conducted DNA methylation profiling in individuals with ASD using various tissues, including post mortem brain tissue, peripheral blood, buccal cells, sperm, placenta, and iPSC-derived organoids. Differentially methylated sites in ASD individuals were identified in genes involved in synaptic function and neuronal development, including *OXTR*, *SHANK3*, *UBE3A*, and *MECP2* [20,21]. However, the exact role of DNA methylation in ASD remains unclear due to ASD’s heterogeneity and the variability of findings across tissues and populations.

Peripheral blood mononuclear cells (PBMCs) have emerged as a practical surrogate for studying DNA methylation in ASD. Although brain tissue is the most relevant sample for investigating neurodevelopmental disorders, PBMCs provide a minimally invasive alternative with demonstrated correlations between blood and brain methylation patterns in specific loci [18,20,22,23]. Moreover, PBMC methylation also shows a correlation with neural activity and ASD-related traits [18,24]. Previous research has highlighted both global and locus-specific alterations in DNA methylation in ASD. For instance, hypomethylation of repetitive elements such as LINE-1 retrotransposons has been observed, reflecting global epigenetic dysregulation [18]. Additionally, hypermethylation of genes like *NCAM1* and *NGF* has been linked to ASD subtypes with distinct clinical features such as neurodevelopmental regression [18,25]. However, it remains unclear whether DNA methylation in the PBMCs of ASD individuals captures sex- and severity-dependent epigenetic patterns that contribute to ASD heterogeneity. Addressing this gap is essential to identify peripheral molecular signatures that may serve as accessible epigenetic marks and to enhance our understanding of the biological basis of ASD heterogeneity.

ASD presents heterogeneity in clinical phenotype and severity, which is reflected in the diverse range of symptoms and impairments observed across individuals with ASD, including variations in social communication, repetitive behaviors, and cognitive abilities. Several factors, including genetic variability, environmental influences, and comorbidities, influence the heterogeneity in ASD severity. For instance, genetic studies have identified numerous genes associated with ASD, many of which contribute to synaptic and neuronal development pathways [26]. Additionally, comorbidities such as attention-deficit/hyperactivity disorder (ADHD), anxiety disorders, and intellectual disability are common in ASD, further contributing to the variability in symptom severity [27]. The Childhood Autism Rating Scale, Second Edition (CARS-2) is a widely used tool for assessing the severity of ASD. It evaluates behaviors across 15 domains related to social communication, emotional regulation, and repetitive behaviors, providing a comprehensive score that reflects the level of impairment. Scores can be categorized into three levels of severity: mild, moderate, and severe. The CARS-2 score captures the heterogeneity of ASD severity by quantifying impairment across symptom domains. The heterogeneity of ASD phenotypes is a critical factor in understanding and managing the disorder. Further research is needed to fully elucidate the complex interactions between genetic, environmental, and comorbid factors that contribute to the diverse clinical presentation of this condition. Stratifying ASD cases by clinical severity may help uncover distinct molecular signatures.

In this study, we performed genome-wide DNA methylation profiling of PBMCs obtained from children diagnosed with ASD and typically developing controls using the Infinium MethylationEPIC v2.0 array. We aimed to identify differentially methylated positions (DMPs) and regions (DMRs) associated with ASD and to determine how these epigenetic signatures related to clinical presentation. To elucidate the biological function of these alterations, we integrated our methylation data with curated ASD-related genes from the SFARI database, extensive pathway enrichment analyses, and cross-tissue/cohort validations against independent post mortem brain and peripheral blood datasets. Through this comprehensive approach, we sought to uncover reproducible and severity-associated methylation changes in PBMCs that reflect the underlying neurodevelopmental pathophysiology and phenotypic heterogeneity of ASD.

## 2. Results

### 2.1. Identification of Genome-Wide DNA Methylation Signatures in the ASD Cohort

**Figure 1:**
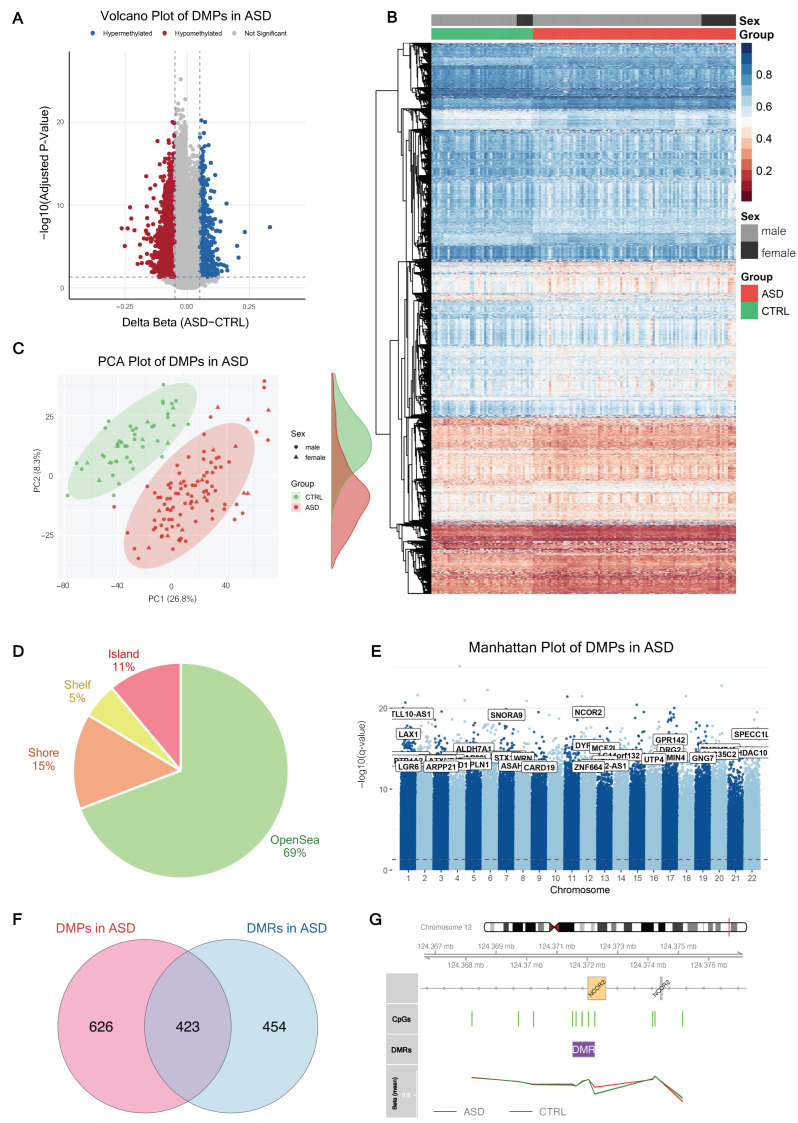
Genome-wide DNA methylation landscape in the PBMCs of children with ASD. (**A**) Volcano plot displaying DMPs between ASD (*n* = 100) and CTRL (*n* = 50). Dashed lines indicate the significance thresholds (|Δβ| > 0.05, adjusted *p*-value < 0.05). Blue points indicate hypermethylated probes, while red points indicate hypomethylated probes. (**B**) Heatmap and unsupervised hierarchical clustering of the significant DMPs. Rows represent individual CpG sites, and columns represent cohort individuals. The color scale indicates the DNA methylation level (beta value). (**C**) Principal Component Analysis (PCA) plot of the study cohort based on significant DMPs. Green and red ellipses represent the 95% confidence intervals for the CTRL and ASD groups, respectively. (**D**) Distribution of DMPs across genomic features. The pie chart illustrates the percentages of identified DMPs located in CpG Islands, Shores, Shelves, and the Open Sea. (**E**) Manhattan plot of DMPs across all chromosomes. The −log10 (q-value) is plotted against the genomic position, dashed line indicate the significance thresholds (adjusted *p*-value < 0.05). (**F**) Venn diagram illustrating the overlap between genes harboring DMPs and those associated with DMRs. (**G**) Representative DMR at the *NCOR2* locus on chromosome 12. The upper panel shows the chromosomal location and gene structure. The middle panel indicates the location of individual CpG probes (green bars) and the identified DMR (purple bar). The bottom panel shows the mean beta-values for the ASD (red line) and CTRL (green line) groups across the region.

To identify the epigenetic landscape associated with ASD, we performed genome-wide DNA methylation profiling on PBMCs from children with ASD (*n* = 100) and age- and sex-matched typically developing controls (CTRL; *n* = 50) using the Infinium MethylationEPIC v2.0 array. Firstly, there were no significant differences in age, sex, and blood-cell-type composition between children with ASD and the CTRL group; demographic information is shown in Supplementary Table S1 and Figure S1. We identified 3507 differentially methylated positions (DMPs) using a linear model adjusted for age, sex, and blood-cell-type composition with adjusted *p*-value < 0.05 and |Δβ| > 5% (Table 1 and Figure 1A). Additionally, 1049 genes harbored these DMPs (Supplementary Table S2). Among the 3507 DMPs identified in the primary cell-type-adjusted model, 2876 probes (82.0%) were hypomethylated, and 631 probes (18.0%) were hypermethylated in ASD. To assess whether this directional bias was influenced by cell-type composition, we compared models with and without estimated cell-type composition adjustment and found highly concordant DMP results and a consistent hypomethylation bias (Figure S2).

Unsupervised hierarchical clustering and PCA of the selected DMPs were used to visualize methylation patterns captured by the differential methylation analysis. Within this DMP-defined feature space, ASD and CTRL samples showed group-related differences (Figure 1B,C). The identified DMPs were non-randomly distributed across the genome. Mapping of these sites relative to CpG islands revealed that the majority (69%) were located in “open sea” regions, while 11% resided within CpG islands, and the remaining 20% were situated in shelf and shore regions (Figure 1D). The chromosomal distribution, visualized in a Manhattan plot, highlighted several high-significance loci, including genes previously implicated in neurodevelopment, such as *NCOR2*, *SNORA9*, and *TTLL10-AS1* (Figure 1E and Table 1).

### 2.2. Regional Differential Methylation Analysis

In addition to individual CpG positions, we identified differentially methylated regions (DMRs) to capture broader epigenetic shifts using the DMRcate package with an FDR < 0.001 and maximum absolute methylation difference (maxdiff) > 5%. We identified 814 DMRs spanning 877 gene regions in the PBMCs of individuals with ASD (Table 2 and Table S3). Among genes harboring DMPs and DMRs, we identified 423 overlapping genes (Figure 1F), including *NCOR2*, *SNORA9*, and *TTLL10-AS1*. A representative example of this convergence was observed at the *NCOR2* locus on chromosome 12, where a cluster of significant CpG sites mapped directly to an identified DMR within the gene (CpG island), showing consistent hypermethylation in the ASD group compared to controls (Figure 1G). These findings indicated that a substantial portion of the ASD-associated epigenetic signature occurs within a coordinated genomic region, potentially affecting transcriptional regulation.

### 2.3. Functional Enrichment and Regulatory Networks of ASD-Associated Epigenetic Signatures

To comprehensively evaluate the biological implications of DNA methylation alterations in PBMCs from the ASD cohort, functional enrichment analysis was performed using the Enrichr platform. We investigated Gene Ontology (GO) domains, including biological processes, cellular components, and molecular functions. To minimize bias and provide a biological context, we focused on both the top nominally significant functions and functions previously implicated in ASD, as shown in Table 3. Within the GO biological process category, the most nominally significant enriched pathway was anterograde trans-synaptic signaling (*p*-value = 9.05 × 10^−4^, adjusted *p*-value = 0.891), alongside strictly metabolic and regulatory terms such as the negative regulation of lipid localization (*p*-value = 1.20 × 10^−3^, adjusted *p*-value = 0.891) and cellular response to EGF stimulus (*p*-value = 2.79 × 10^−3^, adjusted *p*-value = 0.891). The results also reflected processes classically disrupted in ASD. These included chemical synaptic transmission (*p*-value = 4.98 × 10^−3^, adjusted *p*-value = 0.891) and immune and inflammatory cascades, such as the positive regulation of interleukin-1 production (*p*-value = 5.20 × 10^−3^, adjusted *p*-value = 0.891) and the cellular response to TNF (*p*-value = 1.51 × 10^−2^, adjusted *p*-value = 0.891). The GO cellular component analysis also reflected the peripheral immune compartment, such as MHC protein complex (*p*-value = 1.83 × 10^−2^, adjusted *p*-value = 0.970). For GO molecular function, calcium ion binding (*p*-value = 3.40 × 10^−3^, adjusted *p*-value = 0.861) was the most statistically significant enrichment, a critical pathway for both immune cell activation and neuronal excitability. And we also observed enrichment for voltage-gated monoatomic cation channel activity (*p*-value = 4.14 × 10^−2^, adjusted *p*-value = 0.861), a relevant to ASD. Collectively, these results showed that PBMC methylation profiles in ASD may capture a blend of systemic regulatory changes and classic neuroimmune disruptions.

### 2.4. Disease Perturbation Signatures and Transcriptional Regulations of the DMPs in ASD

**Figure 2:**
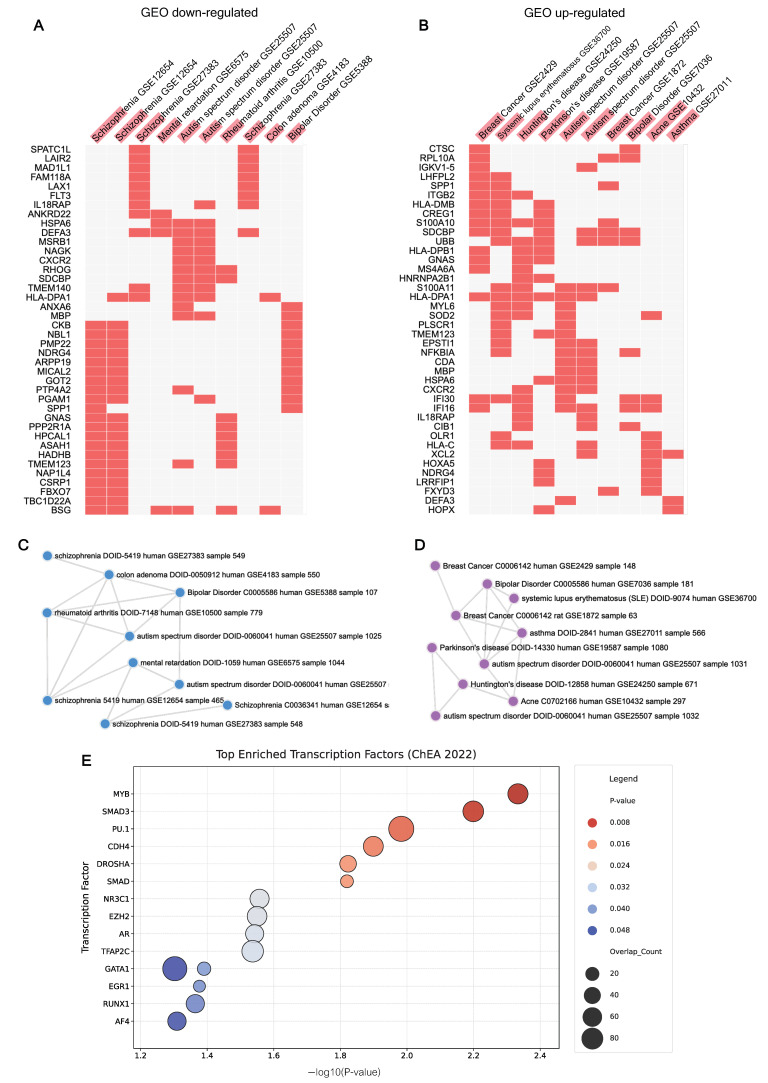
Gene set enrichment and transcriptional regulation analysis of DMPs. Enrichment analyses were performed using the Enrichr platform to identify disease associations and upstream regulators. Heatmaps showed disease associations for genes harboring DMPs, based on Gene Expression Omnibus (GEO) datasets, stratified into (**A**) upregulated and (**B**) downregulated gene sets. Rows represent specific genes, and columns represent associated disease signatures from independent GEO datasets. Network visualization of disease clusters for (**C**) upregulated and (**D**) downregulated gene sets. (**E**) Bubble plots illustrating the top upstream regulator from the ChEA 2022 database, the y-axis displays the transcription factors, color corresponds to the statistical significance of the enrichment, and bubble size corresponds to the number of overlaps.

To determine our epigenetic findings within known transcriptomic disease signatures, enrichment was performed using disease perturbations from the GEO databases (up- and downregulated gene sets) available in Enrichr. These analyses evaluate whether the genes harboring ASD-associated DMPs in our cohort are known to be systematically dysregulated in specific clinical states. Interestingly, the DMP-associated genes showed nominally significant overlapping with gene expression signatures from transcriptomic studies of psychiatric and neurodevelopmental disorders (Figure 2A–D). Within the downregulated gene sets, the most nominally significant signatures were for schizophrenia (*p*-value = 5.77 × 10^−4^, adjusted *p*-value = 0.365) and mental retardation (*p*-value = 1.27 × 10^−2^, adjusted *p*-value = 0.999). Crucially, multiple independent datasets for autism spectrum disorder were identified as top significant terms in both the downregulated and upregulated perturbation libraries (Figure 2A,B) (Supplementary Table S4). These findings suggested that DMP-associated genes overlap with gene sets previously reported to be dysregulated in ASD and related neurodevelopmental or psychiatric conditions.

To determine whether overlapping genes (423 genes harboring both DMPs and DMRs) exhibit distinct functional enrichment compared to non-overlapping genes (unique to DMPs) (Figure 1F), we compared their disease perturbation profiles across GEO datasets. The analysis revealed distinct functional trajectories between the two gene sets. The 423 overlapping genes were predominantly enriched for broader mood and psychiatric conditions, including schizophrenia and bipolar disorder. In contrast, genes unique to DMPs showed highly specific enrichment for ASD and related neurodevelopmental conditions (e.g., mental retardation) (Supplementary Table S4).

In addition to identifying upstream regulatory drivers of the observed DNA methylation alterations, we performed transcription factor (TF) enrichment analysis using the ChEA 2022 database (available in Enrichr). This analysis determines whether genes harboring DMPs are regulated by specific transcription factors, thereby linking epigenetic changes to coordinated gene expression networks. We identified several interesting regulatory hubs (Figure 2E and Supplementary Table S4). Among the top significant enrichments were gene networks regulated by SMAD3 (*p*-value = 6.32 × 10^−3^, adjusted *p*-value = 0.999) and PU.1 (*p*-value = 1.04 × 10^−2^, adjusted *p*-value = 0.999). The significant enrichment of PU.1 targets reinforces the involvement of innate immune and neuroinflammatory pathways in the PBMC methylation signature. Interestingly, a targeted query revealed significant overlap with genes regulated by CDH4 derived from a mouse embryonic stem cell model of autism (*p*-value = 1.26 × 10^−2^, adjusted *p*-value = 0.999). The presence of the specific signature indicated that the differential methylation occurring in our cohort directly intersects with established ASD regulatory networks.

### 2.5. DNA Methylation Profiles in ASD Individuals Stratified by Clinical Behavioral Severity

**Figure 3:**
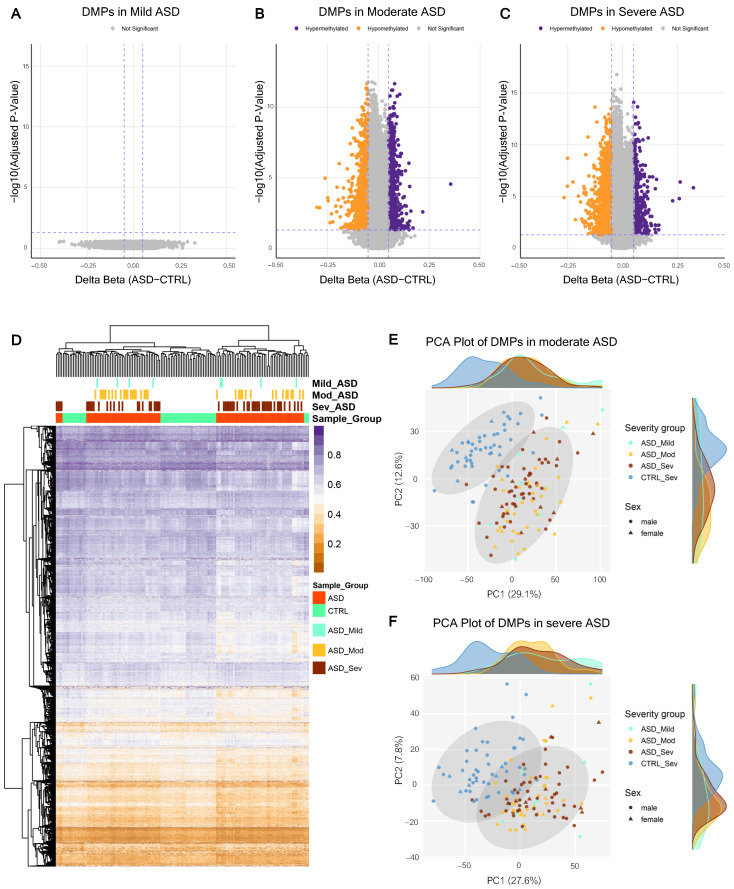
Identification and overlap of Differentially Methylated Positions (DMPs) stratified by ASD clinical severity. (**A**–**C**) Volcano plot of differential methylation analysis comparing mild, moderate, and severe ASD groups against CTRL. Dashed lines indicate the significance thresholds (|Δβ| > 0.05, adjusted *p*-value < 0.05). (**D**) Hierarchical clustering heatmap of the significant DMPs across mild, moderate, and severe ASD individuals, demonstrating overlapping methylation patterns rather than distinct subgroup separation. (**E**) Principal Component Analysis (PCA) plot based on the significant DMPs in the moderate ASD. (**F**) PCA plot based on the significant DMPs in the severe ASD.

To determine whether the DNA methylation alterations were associated with clinical presentation, we stratified the ASD cohort into mild (*n* = 8), moderate (*n* = 36), and severe ASD (*n* = 56) based on CARS-2 scores. We then performed differential methylation analyses comparing each clinical group with sex- and age-matched CTRL. Due to the small sample size (*n* = 8), the mild ASD group was underpowered to detect DMPs; therefore, further interpretation is not possible (Figure 3A). While robust epigenetic alterations were detected in the more symptomatic cohorts, we identified 4173 significant DMPs in the moderate ASD group and 3569 significant DMPs in the severe ASD group (adjusted *p*-value < 0.05 and |Δβ| > 5%) (Figure 3B,C) (Supplementary Table S5). This indicated that DNA methylation changes in peripheral blood were associated with moderate-to-severe clinical presentation.

**Figure 4:**
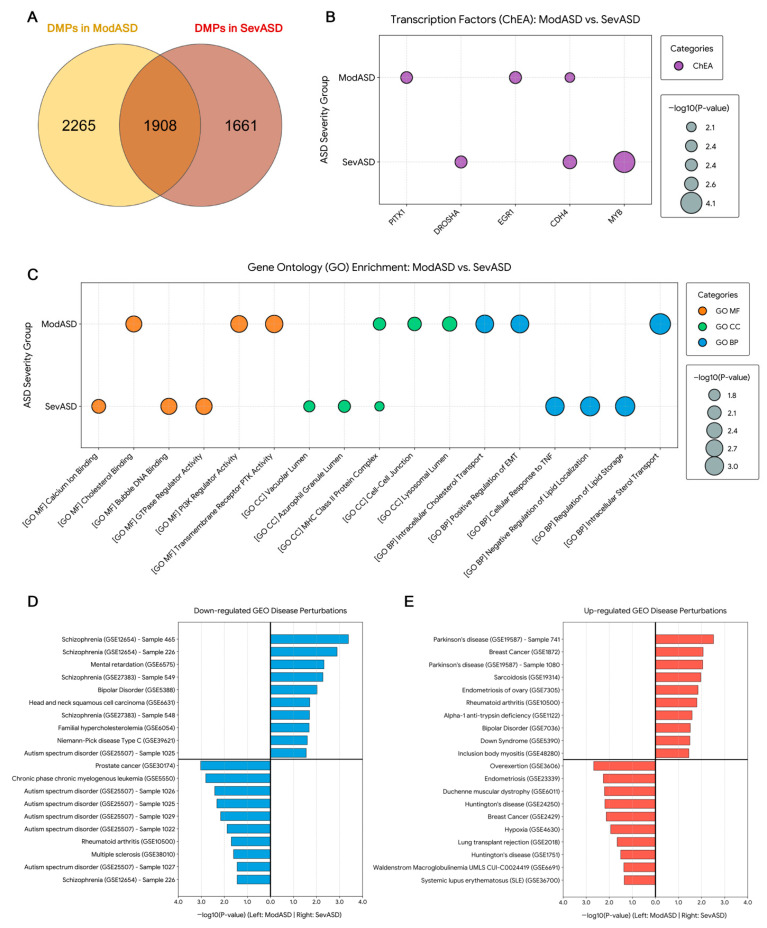
Functional and disease perturbation enrichment profiles of the moderate and severe ASD. (**A**) Venn diagram illustrating the overlapping of DMPs between moderate and severe ASD. (**B**,**C**) Bubble plots illustrating the top enriched terms, x-axis displays the specific enriched terms, and bubble size corresponds to the statistical significance of the enrichment. (**B**) Enrichment of transcription factor targets derived from the ChEA database. (**C**) Gene Ontology enrichment analysis encompassing Biological Processes (GO_BP, blue), Cellular Components (GO_CC, green), and Molecular Function (GO_MF, orange). (**D**,**E**) Bar charts comparing the top 10 significantly enriched GEO disease perturbation signatures for moderate ASD (left) and severe ASD (right). The x-axis represents enrichment significance.

To visualize methylation patterns across ASD severity groups, we performed hierarchical clustering and PCA using the DMPs identified in the severity-stratified analyses (Figure 3D,F). The heatmap and PCA showed partially overlapping methylation patterns between the moderate and severe ASD groups, which indicated that ASD individuals share an underlying baseline of epigenetic dysregulation. However, intersection analysis of the specific probes revealed biological divergence between the moderate and severe ASD groups (Figure 4A). Intersection analysis further identified 1908 shared DMPs between the moderate and severe groups, as well as subgroup-specific DMPs (Figure 4A). These findings suggest that moderate and severe ASD may share a substantial component of ASD-associated methylation alterations while also potentially exhibiting severity-associated differences.

### 2.6. Comparison of Biological Functions Between DMPs in Moderate and Severe ASD

To elucidate the biological differences between moderate and severe ASD, we conducted a comparative gene set enrichment using the Enrichr platform across functional categories: transcription factor binding (ChEA 2022), biological processes (BP), cellular components (CC), and molecular functions (MF). Firstly, the most nominally significant enriched transcription factor targets in the moderate ASD were EGR1 (*p*-value = 0.0038, adjusted *p*-value = 0.999) and PITX1 (*p*-value = 0.0042, adjusted *p*-value = 0.999) (Figure 4B). Severe ASD showed strong enrichment for targets of MYB (*p*-value = 8.08 × 10^−5^, adjusted *p*-value = 0.061) and DROSHA (*p*-value = 0.0038, adjusted *p*-value = 0.713). Interestingly, CDH4 was enriched in both ASD groups (modASD, *p*-value = 0.0074, adjusted *p*-value = 0.999; sevASD, *p*-value = 0.0023, adjusted *p*-value = 0.713), suggesting shared components in neurodevelopmental etiology. Gene Ontology enrichment analysis revealed that both moderate and severe ASD phenotypes shared basic biological functions, particularly immune-related and lipid-metabolic processes (Figure 4C). Notably, both severity groups demonstrated a shared significant enrichment for the MHC Class II protein complex (modASD *p*-value = 0.014, adjusted *p*-value = 0.777; sevASD *p*-value = 0.031, adjusted *p*-value = 0.972) (Figure 4C). Additionally, moderate ASD exhibited distinct enrichment in structural and kinase signaling networks, including cell–cell junctions (*p*-value = 0.0098, adjusted *p*-value = 0.777) and the positive regulation of epithelial to mesenchymal transition (*p*-value = 0.0019, adjusted *p*-value = 0.941), PI3K regulator (*p*-value = 0.0031, adjusted *p*-value = 0.770), and transmembrane receptor protein tyrosine kinase activity (*p*-value = 0.0023, adjusted *p*-value = 0.770). In contrast, severe ASD demonstrated a distinct functional and neuroinflammatory signature, including cellular response to tumor necrosis factor (TNF) (*p*-value = 0.0015, adjusted *p*-value = 0.859), GTPase regulator activity (*p*-value = 0.0037, adjusted *p*-value = 0.812), calcium ion binding (*p*-value = 0.0089, adjusted *p*-value = 0.812), and bubble DNA binding (*p*-value = 0.0039, adjusted *p*-value = 0.812).

Finally, we compared genes harboring DMPs against the GEO disease perturbations database (up- and downregulated gene signatures) using Enrichr. In moderate ASD, the differentially methylated gene set was nominally significantly enriched for autism spectrum disorder transcriptomic profiles and Huntington’s disease (Figure 4D,E), while the severe ASD group exhibited an overlap with severe psychiatric and neurocognitive disorders, including schizophrenia, mental retardation, bipolar disorder, and Parkinson’s disease.

### 2.7. Enrichment of Autism-Candidate Genes in Genes Harboring DMPs

**Figure 5:**
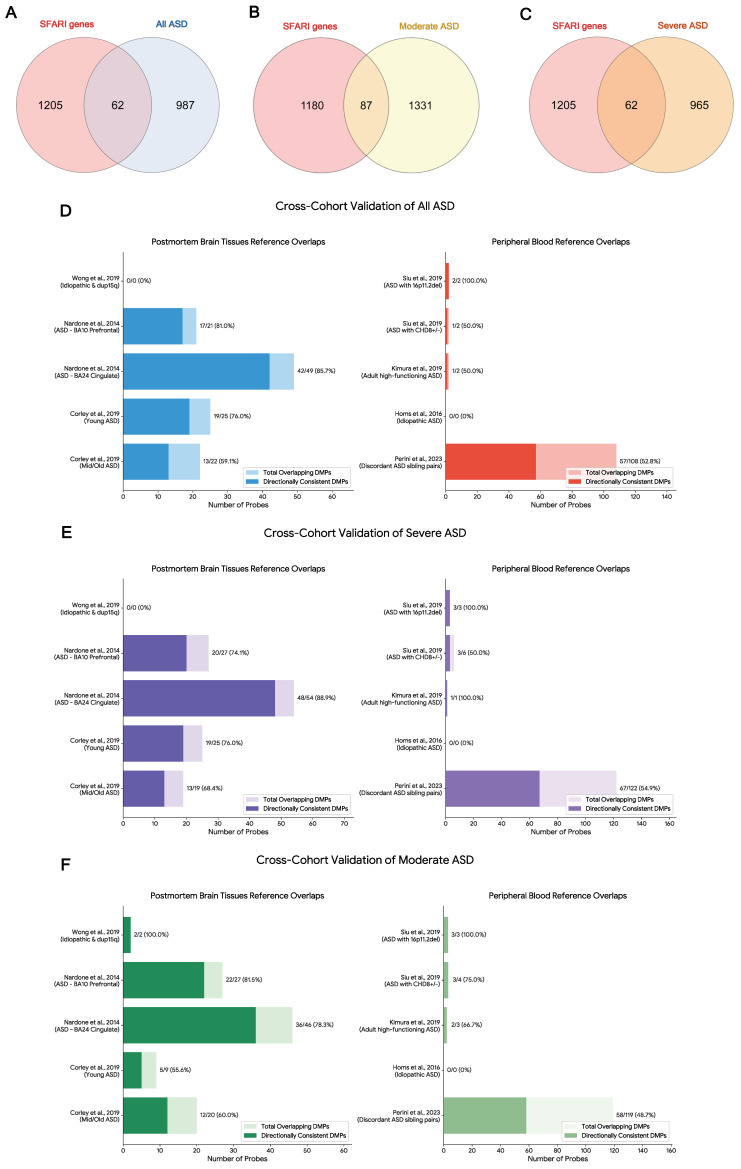
Cross-cohort/tissue validation of epigenetic signatures in the PBMCs of ASD. (**A**–**C**) Venn diagram illustrating the overlapping of SFARI genes and DMPs in all ASD (**A**), moderate ASD (**B**), and severe ASD (**C**). Bar plots represent the overlap of DMPs identified in our ASD cohort against independent reference datasets [28,29,30,31,32,33,34] ((**D**), all ASD; (**E**), moderate ASD; (**F**), severe ASD). Left Panel: Overlaps with post mortem brain tissue signatures. Right Panel: Overlaps with independent peripheral blood signatures. Lighter shaded bars denote the total number of cross-matched probes. Darker-shaded bars represent the subset of overlapping probes showing the same direction of methylation change as observed in our PBMC cohort. Hypergeometric tests were used to evaluate overlap enrichment, and exact binomial tests were used to assess whether directional concordance exceeded a 50% null expectation. Adjusted *p*-values are provided in Supplementary Table S6.

To determine whether genes harboring DMPs are functionally relevant to ASD etiology, we performed a hypergeometric enrichment analysis using the SFARI Gene database. We assessed the overlap of genes harboring DMPs across three analytical groups, including all ASD (1049 genes), moderate ASD (1418 genes), and severe ASD (1027 genes), against various SFARI gene scoring categories (Table 4). We found highly significant enrichment of DMP genes among all SFARI gene categories across all phenotypic groups, with the most robust enrichment observed in the moderate ASD group (87 overlapping genes, adjusted *p*-value < 0.001), followed by severe ASD (62 genes, adjusted *p*-value = 0.001) and all ASD (62 genes, adjusted *p*-value = 0.001) (Table 4 and Figure 5A–C). When dividing the SFARI genes by confidence scores, high-confidence ASD risk genes (Score 1 and Score 2) were consistently enriched across all three ASD groups. Specifically, Score 1 genes were significantly enriched in all ASD (adjusted *p*-value = 0.018), moderate ASD (adjusted *p*-value = 0.018), and severe ASD (adjusted *p*-value = 0.020). Interestingly, when separating syndromic from non-syndromic classifications, non-syndromic genes were significantly overrepresented in all ASD groups. In contrast, significant enrichment for syndromic ASD genes was exclusively observed in the severity-stratified groups (moderate ASD: *p* = 0.010; severe ASD: *p* = 0.028), but not when analyzing the combined all ASD cohort. Moreover, severe ASD was uniquely enriched for Score 3 genes (adjusted *p*-value = 0.046).

### 2.8. Cross-Cohort and Cross-Tissue Validation of ASD Epigenetic Signatures

A major challenge in peripheral-blood-based epigenetic studies of neurodevelopmental disorders is determining whether systemic signatures reflect pathologically relevant changes in the central nervous system. To address this, we performed a cross-cohort validation by overlapping our identified DMPs with independent microarray datasets from both peripheral blood and post mortem brain tissue (Supplementary Table S6). Interestingly, our PBMC-derived DMPs showed significantly enriched and directional concordance with post mortem brain tissue methylomes. When cross-validating against the anterior prefrontal cortex (BA10) and anterior cingulate cortex (BA24) datasets from Nardone et al., 2014 [31], we observed significant hypergeometric overlap across all severity subgroups (hypergeometric adjusted *p*-value < 0.05). Notably, these shared probes demonstrated directional concordance, particularly in the BA24 region for the severe ASD group, where 88.9% of the 54 overlapping probes were directionally consistent (binomial *p*-value = 3.26 × 10^−9^ and adjusted *p*-value = 7.82 × 10^−8^) (Figure 5D–F) [31]. Furthermore, overlapping with the age-stratified cohort (Corley et al., 2019), our PBMC signatures aligned strongly with the post mortem brain of the young cohort, such as in all ASD with 25 overlaps at a 76% consistency rate (hypergeometric *p*-value = 4.45 × 10^−15^, adjusted *p*-value = 5.34 × 10^−14^), however, directional consistency across these age-stratified groups did not reach statistical significance (binomial *p*-value = 0.015 and adjusted *p*-value = 0.050) [28].

To validate our findings against independent peripheral blood cohorts, we cross-validated our data with known syndromic ASD mutations (Siu et al., 2019) and a large-scale EPIC array cohort (Perini et al., 2023) [30,32,33]. Significant enrichment of shared genomic loci was observed in the moderate and severe ASD groups when compared with the syndromic ASD signatures reported by Siu et al., 2019 [33]. (hypergeometric adjusted *p*-value < 0.05). By contrast, although our DMPs showed the largest absolute number of shared loci with the large-scale EPIC array sibling cohort from Perini et al., 2023 [32], these overlaps did not exceed chance expectations (hypergeometric adjusted *p*-value > 0.90). Similarly, directional concordance with independent peripheral blood cohorts was not statistically significant based on binomial testing (Figure 5D–F).

Among these DMPs, several key neurodevelopmental genes were identified, including two distinct hypomethylated probes mapping to *CNTNAP2* (cg05640346 and cg14859916), as well as *GRIN1* and *CUX1* (Table 5). Furthermore, comparisons against syndromic ASD blood cohorts (Siu et al., 2019) [33] identified shared epigenetic dysregulation, highlighted by the consistent hypermethylation of *NCOR2* across our ASD cohort and the 16p11.2 deletion reference group. Remarkably, several PBMC signatures also showed directional concordance with post mortem brain tissue methylomes. Cross-validation against Nardone et al. and Corley et al. [28,31] identified consistent hypomethylation in genes such as *PACSIN1*, *WDR70*, and *FAM110D*. Most notably, the probe cg09384035, which maps to the long non-coding RNA (*LINC01181*), demonstrated reproducibility. This locus was consistently hypomethylated across all three of our severity subgroups and replicated in both an independent peripheral blood cohort (Perini et al., 2023) [32] and a post mortem anterior cingulate cortex dataset (Nardone et al., BA24) [31], representing a reproducible cross-tissue epigenetic signature in ASD. These findings indicated that our DMPs in ASD PBMCs represented epigenetic signatures that were reproducible across different tissues and independent cohorts, showing directional concordance with known neurodevelopmental risk factors.

## 3. Discussion

Although DNA methylation profiles in PBMCs do not fully reflect brain epigenetic states, blood–brain correlations at specific loci support using blood as a peripheral marker for human brain research [22,23,35]. Our study identifies a distinct epigenetic signature in the PBMCs of children with ASD, characterized by robust and reproducible DMPs associated with clinical presentation. Specifically, we identified a genome-wide landscape comprising 3507 DMPs and 814 DMRs. The convergence of these alterations at specific loci, such as *NCOR2*, suggested that these findings represent coordinated regional methylation perturbations rather than isolated probe-level artifacts. A notable feature of the ASD-associated methylation signature was a strong hypomethylation bias, with approximately 82% of DMPs. This directional pattern remained essentially unchanged when comparing the primary model, which included stringent adjustment for estimated immune-cell proportions, with a model without cell-type covariates (Figure S2). This suggested that the hypomethylation bias is unlikely to be explained solely by estimated differences in blood-cell-type composition. Nevertheless, because cell-type proportions were computationally inferred, this finding should be interpreted cautiously. Future studies using purified PBMC subsets or single-cell methylation profiling are warranted to determine whether this directional asymmetry reflects specific immune-cell populations or broader systemic epigenetic dysregulation in ASD.

Our functional enrichment analyses identified representation of neuronal and synaptic terms within a peripheral immune cell compartment, including anterograde trans-synaptic signaling and chemical synaptic transmission. The brain–blood finding in epigenetic data may have two possible explanations. First, DNA methylation patterns that are formed early in development may be partly shared across different tissues. Therefore, methylation changes seen in blood may reflect similar changes in the developing brain [22,36]. Second, immune cells and neurons share many receptors and signaling pathways. Changes in these pathways in the PBMCs may influence how immune cells communicate with or move toward the central nervous system [37,38]. Consistent with the well-documented immune components of ASD pathogenesis, our analysis revealed significant inflammatory and immune-regulatory pathways. The data highlighted the MHC protein complex and the azurophil granule lumen, alongside the positive regulation of interleukin-1 (IL-1) production. Elevated levels of pro-inflammatory cytokines, particularly IL-1 and TNF, are consistently reported in the serum and post mortem brain tissue of individuals with ASD [39,40,41]. Our findings raise the possibility that PBMC methylation differences may be linked to immune-regulatory pathways in ASD, although functional studies are required to establish mechanistic relevance.

Moreover, the comparison of our epigenetic signatures of ASD with known transcriptomic disease signatures provided compelling evidence reflecting disease association. The significant enrichment of our DMP-associated genes in the dysregulated transcriptomic signatures of ASD, schizophrenia, and mental retardation validated the hypothesis that peripheral tissues capture core gene regulatory networks traditionally associated with the central nervous system. Additionally, transcription factor enrichment analysis identified PU.1 and SMAD3 as significantly enriched regulatory hubs, which suggests a potential role for chronic immune dysregulation. PU.1 is a master transcription factor required for the differentiation and activation of myeloid cells, including circulating monocytes and CNS microglia, as well as other immune cells [42,43,44]. Epigenetic changes, together with SMAD3, a key protein in the TGF-beta signaling pathway, suggest that immune cells in individuals with ASD may be in a continuously dysregulated state [45,46,47]. In addition to immune dysregulation, our transcriptional regulatory analysis revealed core neurodevelopmental alterations, most notably the significant enrichment of CDH4 target genes. Cadherin-4, or R-cadherin (CDH4) is a cell adhesion molecule that regulates early brain development, specifically neuronal migration, axon guidance, and the structural establishment of synaptic circuits [48]. The disruption of synaptic adhesion and neuronal scaffolding is a leading pathophysiological model for ASD [49].

The comparative gene set enrichment analysis of moderate and severe ASD individuals revealed a molecular landscape characterized by both shared neurodevelopmental disruptions and distinct pathways. Interestingly, both ASD cohorts exhibited significant enrichment for CDH4 targets and the MHC Class II protein complex. This underscores fundamental deficits in neuronal connectivity that are core features of ASD regardless of severity. Furthermore, the mutual enrichment of MHC Class II components reinforces the well-documented hypothesis that neuroimmune crosstalk and immune activation play foundational roles in the etiology of ASD [50]. The molecular terms unique to moderate ASD strongly point toward alterations in synaptic plasticity and localized cellular metabolic pathways. In contrast, severe ASD exhibited an enrichment profile for neuroinflammation, such as a prominent cellular response to tumor necrosis factor (TNF).

Our hypergeometric distribution analysis provides evidence that differential DNA methylation in ASD is not randomly distributed across the genome but rather concentrated within genetic risk loci. By overlapping our DMP-associated genes with the SFARI Gene database, we demonstrated a consistent overrepresentation of DMPs within highly confident (Score 1) ASD candidate genes across all ASD and severity groups. An interesting finding from our severity-stratified analysis is the divergence in enrichment profiles between syndromic and non-syndromic genes. When the ASD cohort was classified by symptom severity, significant enrichment for syndromic and highly confident genes was found uniquely in the moderate and severe ASD groups. These findings suggested that methylation differences may be associated with symptom severity, but causal relationships cannot be inferred from the present cross-sectional design [25,51,52,53,54].

Cross-cohort comparisons provided exploratory support for selected ASD-associated methylation loci across independent datasets, although the strength of overlap and directional concordance varied by tissue type, cohort, and platform. By comparing our DMPs with independent post mortem brain and peripheral blood datasets, we identified selected candidate loci with cross-dataset overlap or directional consistency. Notably, we identified reproducible hypomethylation loci mapping to Contactin Associated Protein-like 2 (*CNTNAP2*) in both our PBMC cohort and an independent ASD peripheral blood cohort. *CNTNAP2* is among the best-characterized autism-susceptibility genes in the literature, playing an essential role in neuronal development, synaptogenesis, and neuronal migration [55,56]. We also found overlapping in crucial synaptic and neuronal genes, including Glutamate Ionotropic Receptor NMDA Type Subunit 1 (*GRIN1*) and Cut-Like Homeobox 1 (*CUX1*) genes. Furthermore, our comparison with the syndromic ASD cohort revealed a consistent hypermethylation of Nuclear Receptor Corepressor 2 (*NCOR2*) in ASD with 16p11.2 deletion. NCOR2 is a critical transcriptional corepressor that modulates chromatin structure and has been implicated in memory consolidation and neurological function [57]. The common epigenetic dysregulation of *NCOR2* between our idiopathic ASD cohort and a known genetic deletion syndrome highlights a potential convergent epigenetic pathway linking distinct genetic architectures to a shared systemic ASD phenotype. Importantly, directional concordance between PBMCs and brain tissue does not imply causation, identical cell-specific regulation, or direct equivalence between peripheral immune cells and neuronal cell populations. These findings should therefore be interpreted as exploratory cross-dataset support for selected candidate loci.

PBMCs serve as a practical surrogate tissue for studying systemic epigenetic alterations in ASD, given their accessibility and established correlations with methylation of brain tissue [22,23]. While the use of PBMCs provides a minimally invasive approach, further validation in neuronal models is necessary to establish the functional relevance of the identified DMPs in neurodevelopmental pathways [21,34]. Integrating multi-omics approaches, such as transcriptomics and proteomics, could further elucidate the downstream effects of these methylation changes. Despite the strengths of our study, several variations must be acknowledged. The sample size, while sufficient to detect significant DMPs, remains relatively modest compared to large-scale ASD epigenome-wide studies [25,51,58,59]. Additionally, environmental influences, genetic background, and medication use were not fully controlled, which may contribute to the observed variability in methylation patterns. Future studies with larger, ethnically diverse cohorts and longitudinal designs will be crucial in validating these findings and assessing the methylation signatures over time [18,51].

The lack of DMPs in the mild group is likely due to the limited sample size restricting statistical power, rather than mild ASD lacking an epigenetic signature. Another limitation of this study is the unbalanced case–control ratio, with 100 ASD participants and 50 typically developing controls. Future studies using larger, independent, and more balanced cohorts are therefore warranted to validate the ASD-associated PBMC methylation signatures identified in this study. Although several GO terms and disease-associated gene sets showed nominal enrichment, most did not remain significant after correction for multiple testing, likely reflecting the large number of gene sets evaluated in the enrichment analyses. Therefore, these findings should be interpreted as exploratory and hypothesis-generating, providing biological context for ASD-associated DMP genes rather than definitive evidence of pathway-level dysregulation.

Additionally, while methylation profiles in PBMCs offer valuable insights, they may not fully capture epigenetic changes in individuals’ brains. Future studies integrating PBMCs and brain tissue epigenetic data would help validate the systemic relevance of our findings. Lastly, functional validation of identified DMPs and their roles in ASD-associated pathways is necessary to establish causal relationships and potential molecular mechanisms.

## 4. Materials and Methods

### 4.1. Participant Recruitment

We performed a case–control study of Thai children (≤18 years) with autism spectrum disorder (ASD) and typically developing controls (CTRL); the study protocol was approved by the Institutional Review Board of the Faculty of Medicine, Chulalongkorn University, Bangkok, Thailand (COA No. 1238/2022; date of approval: 24 August 2022, COA No. 1153/2023; date of approval: 24 August 2023, and COA No. 1172/2024; date of approval: 24 August 2024). All participants were recruited and evaluated at the Division of Growth and Development, Department of Pediatrics, Faculty of Medicine, Chulalongkorn University. ASD cases were diagnosed by developmental pediatricians and clinical psychologists using the Diagnostic and Statistical Manual of Mental Disorders, Fifth Edition (DSM-5) criteria with supporting Childhood Autism Rating Scale, Second Edition (CARS-2) assessment together with detailed clinical histories including age at first concern, developmental milestones, comorbidities, current medications and therapies; cases underwent age-appropriate cognitive/developmental testing (Mullen Scales of Early Learning, Wechsler Preschool and Primary Scale of Intelligence-Fourth Edition (WPPSI-IV), Wechsler Abbreviated Scale of Intelligence-Second Edition (WASI-II)). Exclusion criteria included lack of parental consent and developmental delay attributable to known causes (e.g., childhood disintegrative disorder, syndromic/genetic conditions such as Down or Rett syndromes, obvious congenital malformations, neurological disorders with known causes, major psychiatric comorbidity, significant perinatal insults or teratogenic exposures, congenital hypothyroidism, or severe environmental deprivation). Typically developing participants, including full biological siblings of cases who did not meet the ASD criteria and unrelated TD children, were screened with age-appropriate tools (Modified Checklist for Autism in Toddlers (M-CHAT) for 18 months–4 years or Pervasive Developmental Disorders Screening Questionnaire (PDDSQ) for ≥4 years) and, if screening exceeded cut-offs, were further evaluated with CARS-2. Typically developing controls were eligible only if clinical assessment and screening indicated typical neurodevelopment.

Symptom severity was quantified using the CARS-2 assessment. Participants were stratified by CARS-2 total score into the following categories: minimal-to-no symptoms (mild), 15–29.5; mild-to-moderate symptoms (moderate), 30–36.5; and severe symptoms, ≥37. To establish the control group, we employed a frequency matching approach. Typically developing individuals were selected from the enrolled volunteers to match the ASD cohort by biological sex and age at the time of sample collection. We statistically verified that the final cohorts were demographically comparable, with no significant differences in age or sex distributions prior to downstream analysis. Investigators obtained written parental/legal guardian consent and, when feasible, age-adapted assent from children (simplified information and pictorial aids were used for children with a developmental level equivalent to 7–12 years). A single 6 mL peripheral blood sample was collected into an EDTA tube.

### 4.2. Peripheral Blood Mononuclear Cell (PBMC) Isolation

PBMCs were isolated from EDTA whole blood obtained from ASD (*n* = 100) and TD (*n* = 50) children using a density gradient centrifugation method according to the standard procedure of HiSep™ LSM 1077 (HiMedia, Mumbai, India). Briefly, EDTA whole blood was diluted 1:1 with phosphate-buffered saline (PBS, pH 7.4) and then carefully layered over the HiSep™ LSM solution (2 mL) in a sterile 15 mL conical tube. The samples were centrifuged at 1000× *g* for 30 min at room temperature without a brake. Following centrifugation, the PBMC layer was carefully aspirated, transferred to a new tube, and washed twice with PBS by centrifuging at 300× *g* for 10 min to remove HiSep™ LSM and reduce platelet contamination. The resulting cell pellet was counted and resuspended in GENEzol™ Reagent (Geneaid Biotech, New Taipei, Taiwan) before being stored at −80 °C.

### 4.3. DNA Extraction

Genomic DNA was extracted from the PBMCs using GENEzol™ Reagent (Geneaid Biotech, New Taipei, Taiwan) according to the manufacturer’s protocol. Briefly, chloroform was added to the homogenate (200 µL per 1 mL of GENEzol reagent), and the mixture was shaken for 15 s, then incubated at room temperature for 2–3 min. Phase separation was performed by centrifugation at 12,000× *g* for 15 min at 4 °C. The upper aqueous phase, which contained RNA, was carefully removed. Absolute ethanol was then added to the remaining interphase and organic phase, followed by centrifugation at 12,000× *g* for 10 min to precipitate the DNA pellet. The DNA pellet was washed twice with 75% ethanol, air-dried, and dissolved in nuclease-free water.

### 4.4. DNA Methylation Profiling by Infinium MethylationEPIC v2.0 Array

To investigate epigenetic signatures in PBMCs from individuals with ASD, we performed genome-wide DNA methylation profiling of children with ASD (*n* = 100) compared with sex- and age-matched typically developing controls (*n* = 50). Genomic DNA was assessed for quality and integrity prior to array processing. DNA concentration was quantified using a Qubit™ 4.0 Fluorometer with the Qubit™ dsDNA High Sensitivity (HS) Assay Kit (Thermo Fisher Scientific, Waltham, MA, USA). Bisulfite conversion of genomic DNA was performed using the EZ DNA Methylation Kit (Zymo Research, Irvine, CA, USA) according to the manufacturer’s instructions. Genome-wide DNA methylation levels were determined using the Illumina Infinium MethylationEPIC v2.0 array, which interrogates 930,000 CpG sites across the genome, covering promoters, enhancers, and other regulatory elements. This high-resolution platform enabled comprehensive analysis of differentially methylated positions (DMPs) and regions (DMRs). Arrays were scanned using the Illumina iScan System, and raw signal intensity data (IDAT files) were extracted using Illumina GenomeStudio software (v.2011.1).

### 4.5. Data Processing

Raw data (IDAT files) from the Infinium MethylationEPIC v2 arrays were processed in R (v4.4.1) using the minfi R package (v1.50.0) [60]. The .idat files were imported, and quality control (QC) was performed to identify and exclude poor-quality samples based on detection *p*-values. Normalization was performed using the single-sample Noob (ssNoob) method to account for technical variability [61]. Probes with a detection *p*-value > 0.01 were filtered out, and additional filtering steps were applied to remove probes containing single-nucleotide polymorphisms (SNPs) and those located on sex chromosomes (X and Y). Principal component analysis (PCA) was conducted on the normalized dataset to examine sample clustering and potential sources of variance. Scree plots were generated to assess the proportion of variance explained by each principal component. To address batch effects, the ComBat function from the sva package (v3.52.0) was used to normalize data across arrays and slides [62]. Post-normalization, PCA was performed to confirm the effectiveness of batch-effect correction.

### 4.6. Blood-Cell-Type Composition Estimation

To account for the cellular heterogeneity of isolated PBMCs, cell-type proportions were estimated using the Epigenetic Dissection of Intra-Sample-Heterogeneity (EpiDISH) package (v2.20.1) [63]. Firstly, genomic loci represented by multiple redundant probes on the EPIC v2 array were collapsed by calculating the mean intensity for each unique locus. Specifically, the robust partial correlation method was applied to the collapsed beta-value matrix. We utilized the cent12CT.m reference matrix, which provides DNA methylation signatures for major leukocyte subtypes. The resulting cell-type fractions (e.g., neutrophils, B-cells, CD4+ T-cells, CD8+ T-cells, monocytes, NK cells, and eosinophils) were exported for use as covariates in downstream analyses.

### 4.7. Differentially Methylated Positions and Regions

Differentially methylated positions (DMPs) were identified using the limma R package (v3.60.6), which applies linear modeling with empirical Bayes moderation to stabilize variance estimates [64]. Differential methylation analysis was performed using linear models adjusted for age, sex, and estimated blood-cell-type composition. Estimated proportions of major immune cell subsets, including CD4+ T-cells, CD8+ T-cells, NK cells, monocytes, and granulocytes, were included as covariates in the model to control for potential confounding by blood cell heterogeneity. B-cell proportion was not included as an independent covariate to avoid multicollinearity. Methylation M values were used as input, and the model incorporated group differences while adjusting for these covariates. Statistical significance was determined using adjusted *p*-values (e.g., false discovery rate (FDR)) to account for multiple testing. DMPs were defined as probes that met specified thresholds for both statistical significance and biological relevance, such as a minimum difference in methylation levels between groups. Significant DMPs were defined as those with an adjusted *p*-value < 0.05 after correction for multiple testing using the Benjamini–Hochberg method and an absolute delta beta-value (|Δβ|) > 5%. Data visualizations and statistical plots were generated using the R statistical computing environment and the ggplot2 package (v4.0.2) [65].

### 4.8. Differentially Methylated Region (DMR) Identification

DMRs were identified using the DMRcate R package (v3.0.10), which employs a sliding window approach to detect genomic regions containing clusters of nearby CpG sites that exhibit consistent methylation differences between groups [66]. First, t-statistics were calculated for each CpG site to assess differential methylation. These probe-level statistics were then aggregated across genomic regions using a kernel smoothing approach, which gives higher weight to CpGs located closer together. Regions with significant smoothed scores were determined using a false discovery rate (FDR) threshold to correct for multiple testing (0.001). Subsequently, DMRs were identified using the dmrcate function with lambda = 1000 (a smoothing window of 1000 bp) and C = 2 (a scaling factor for Gaussian kernel smoothing). The results were visualized using genomic heatmaps and regional methylation plots to illustrate methylation patterns across identified regions.

### 4.9. Functional Enrichment Analysis, Overlapping DMPs with SFARI Genes, and Independent Datasets

Differentially methylated positions (DMPs) identified in ASD individuals were analyzed using Enrichr [67]. The list of gene-harboring DMPs in ASD individuals was uploaded to the Enrichr tool. The analyses were performed for Gene Ontology, the ChEA 2022 database, and disease perturbation (GEO up- and downregulated dataset). Nominal *p*-values were used for exploratory prioritization, while adjusted *p*-values were reported and used to evaluate multiple-testing-corrected significance. Transcription factor enrichment analysis was performed using ChEA 2022, a comprehensive library of experimentally validated TF–target interactions from the Enrichr platform, to identify regulatory drivers associated with the identified DMPs [68]. To assess the overlap between identified DMP-associated genes and autism-related genes, we overlapped our DMP gene list with the SFARI Gene database (https://gene.sfari.org/), a curated repository of autism risk genes [69]. Genes overlapping in both datasets were visualized using a Venn diagram, created in R using the VennDiagram package (v1.8.2).

Cross-cohort overlap was statistically evaluated using hypergeometric tests. For each comparison, the background universe was defined as the number of CpG probes shared between our EPIC v2.0 dataset and the corresponding reference dataset/platform. Directional concordance among overlapping probes was assessed using exact binomial tests against a 50% null expectation. *p*-values from both hypergeometric and binomial tests were adjusted across comparisons using the Benjamini–Hochberg procedure. Data visualization and figure generation were performed in Python (v3.10) using the Matplotlib (v3.8.2) library [70].

## 5. Conclusions

In conclusion, this study identifies ASD-associated DNA methylation alterations in PBMCs and highlights candidate epigenetic loci related to clinical severity and ASD-relevant gene networks. Cross-cohort analyses provided partial support for selected loci, but concordance varied across datasets, and peripheral blood findings should not be interpreted as direct surrogates of brain methylation states. Future studies in larger, independent cohorts, ideally integrating PBMCs, whole blood, brain tissue, and functional multi-omics data, are needed to validate these candidate methylation signatures and clarify their biological relevance.

## Figures and Tables

**Table 1:** Top genes harboring differentially methylated CpG sites (DMPs) in the PBMCs of ASD compared to CTRL individuals. List of top genes harboring significant DMPs ranked by adjusted *p*-value. Columns show Illumina probe ID, log fold change (logFC), adjusted *p*-value (FDR), mean β-values in ASD and CTRL groups (beta ASD, beta CTRL), methylation difference (Δβ), island context, and annotated gene name. Positive logFC/Δβ indicates hypermethylation in ASD; negative values indicate hypomethylation.

Probe ID	logFC	adj.*p*.val	Beta ASD	Beta CTRL	Delta Beta	Island	Gene Symbol
cg08276543_BC11	0.619	5.93 × 10^−21^	0.865	0.809	0.056	Island	*NCOR2*
cg19724260_BC21	−0.592	9.50 × 10^−21^	0.792	0.852	−0.060	Open Sea	*TTLL10-AS1*
cg18205465_TC11	−0.989	1.31 × 10^−20^	0.070	0.122	−0.053	Island	*SNORA9*; *SNHG15*
cg21785750_TC21	−0.875	1.89 × 10^−18^	0.641	0.762	−0.120	Open Sea	*TRBV28*
cg06044672_TC21	−0.314	3.00 × 10^−18^	0.634	0.688	−0.054	Open Sea	*LAX1*
cg08267167_TC11	−0.663	3.12 × 10^−18^	0.173	0.248	−0.074	Island	*SPECC1L*; *SPECC1L-ADORA2A*
cg22930206_TC21	0.344	1.54 × 10^−17^	0.617	0.560	0.057	Open Sea	*GPR142*
cg09418321_BC21	−0.401	7.10 × 10^−17^	0.703	0.762	−0.059	Open Sea	*DYRK4*
cg11018006_TC21	0.362	1.07 × 10^−16^	0.615	0.554	0.061	Open Sea	*RNU6-611P*
cg26660457_BC11	−0.614	1.33 × 10^−16^	0.126	0.177	−0.052	Island	*CRAMP1*
cg00841725_BC11	−0.380	1.63 × 10^−16^	0.244	0.296	−0.052	Shore	*MCF2L*
cg20391852_BC21	0.397	2.00 × 10^−16^	0.744	0.689	0.055	Shore	*ALDH7A1*
cg13972460_BC11	−0.541	2.53 × 10^−16^	0.388	0.477	−0.089	Island	*TMEM74B*
cg20001152_TC21	0.434	2.73 × 10^−16^	0.736	0.675	0.061	Shore	*DRG2*
cg16927872_BC11	−0.515	6.10 × 10^−16^	0.205	0.268	−0.063	Island	*HDAC10*; *TUBGCP6*
cg25322634_BC11	−0.518	9.25 × 10^−16^	0.279	0.354	−0.075	Shore	*SLC35C2*
cg11822388_TC21	−0.878	1.22 × 10^−15^	0.687	0.798	−0.111	Open Sea	*TRBV28*
cg02325318_BC11	−0.596	1.35 × 10^−15^	0.154	0.212	−0.058	Island	*C14orf132*
cg00702729_BC21	1.492	1.93 × 10^−15^	0.948	0.879	0.070	Shore	*GAL3ST4*; *TRAPPC14*
cg17371350_BC11	−0.516	2.32 × 10^−15^	0.202	0.264	−0.062	Island	*GEMIN4*; *DBIL5P*
cg21095229_BC11	−0.494	2.34 × 10^−15^	0.194	0.250	−0.056	Island	*DENND1B*
cg01804343_TC11	−0.671	2.46 × 10^−15^	0.122	0.176	−0.054	Shore	*STX1A*
cg18813158_BC11	−0.562	2.47 × 10^−15^	0.127	0.177	−0.050	Island	*NKX6-1*
cg12371105_TC11	−0.705	2.57 × 10^−15^	0.143	0.210	−0.066	Island	*SAP30L*; *SAP30L-AS1*
cg27109030_BC11	−0.550	2.81 × 10^−15^	0.226	0.299	−0.073	Shore	*GNG7*
cg21608195_BC11	−0.565	2.97 × 10^−15^	0.162	0.221	−0.059	Shore	*WRN;PURG*
cg04501436_BC21	0.486	3.01 × 10^−15^	0.821	0.770	0.051	Open Sea	*KIF21A*
cg09986774_BC11	−0.679	4.12 × 10^−15^	0.128	0.187	−0.059	Island	*UTP4*; *CHTF8*; *DERPC*
cg07032342_BC21	−0.266	4.48 × 10^−15^	0.632	0.682	−0.050	Open Sea	*SPNS3*;
cg06634060_BC11	−0.580	5.01 × 10^−15^	0.191	0.257	−0.067	Island	*FGF12-AS2*
cg09373727_BC21	−0.349	6.08 × 10^−15^	0.663	0.714	−0.051	Shore	*PTP4A2*
cg25506386_BC11	−0.484	6.10 × 10^−15^	0.187	0.241	−0.053	Island	*ATXN7*; *THOC7*
cg05107860_BC21	0.343	6.33 × 10^−15^	0.662	0.609	0.053	Open Sea	*REV3L*
cg12861503_TC21	−0.287	7.64 × 10^−15^	0.572	0.624	−0.052	Shore	*NEK3*
cg13939546_TC21	−0.670	9.45 × 10^−15^	0.768	0.837	−0.069	Open Sea	*TRBV28*

**Table 2:** Differentially Methylated Regions (DMRs) in the PBMCs of all Individuals with ASD. This table presents the top 30 overlapping DMR genes identified in PBMCs from all individuals with ASD. Columns include chromosome location (seqnames), start and end positions of the DMRs, the number of CpG sites within each region (no.CpG), and the harmonic mean false discovery rate (HMFDR). The maximum and mean methylation differences (maxdiff and meandiff) indicate the extent of methylation change, with negative values representing hypomethylation and positive values representing hypermethylation in ASD samples. The final column lists genes that overlap with or are in proximity to the DMRs.

Seqnames	Start	End	No.CpG	HMFDR	Maxdiff	Meandiff	Overlapping.Genes
chr12	124371516	124372245	5	1.28 × 10^−21^	0.057	0.011	*NCOR2*
chr1	1179199	1180540	7	2.64 × 10^−21^	−0.060	−0.017	*TTLL10*, *TTLL10-AS1*
chr7	44985332	44986984	10	5.04 × 10^−21^	−0.054	−0.011	*SNHG15*, *SNORA9*
chr22	24270502	24271524	8	1.02 × 10^−18^	−0.074	−0.017	*SPECC1L*, *SPECC1L-ADORA2A*
chr12	4589151	4591368	5	1.47 × 10^−17^	−0.053	−0.025	*DYRK4*
chr20	1184421	1185685	6	5.93 × 10^−17^	−0.091	−0.017	*PSMF1*, *TMEM74B*
chr6	36386455	36388467	19	7.98 × 10^−17^	−0.070	−0.007	*ETV7-AS1*, *ETV7*
chr13	112999921	113002110	18	9.43 × 10^−17^	−0.052	−0.031	*MCF2L*
chr16	1610573	1615093	29	1.11 × 10^−16^	−0.070	−0.008	*CRAMP1*, *IFT140*
chr17	18086518	18088266	13	1.27 × 10^−16^	0.062	0.006	*DRG2*
chr22	50244356	50245625	12	2.41 × 10^−16^	−0.063	−0.005	*TUBGCP6*, *HDAC10*, *MAPK12*
chr7	100160031	100160177	4	3.19 × 10^−16^	0.072	0.016	*GAL3ST4*
chr8	477703	478380	2	4.34 × 10^−16^	−0.070	−0.060	*FBXO25*
chr20	46363946	46365815	18	4.90 × 10^−16^	−0.076	−0.010	*AL133227.1*, *SLC35C2*
chr14	96038959	96040064	14	5.94 × 10^−16^	−0.060	−0.007	*C14orf132*
chr7	73719068	73720505	11	8.37 × 10^−16^	−0.056	−0.008	*STX1A*
chr5	154445503	154447000	12	9.44 × 10^−16^	−0.068	−0.007	*SAP30L*, *SAP30L-AS1*
chr17	76725669	76728129	23	9.48 × 10^−16^	−0.053	−0.009	*METTL23*, *JMJD6*
chr1	31938868	31939963	4	1.07 × 10^−15^	−0.052	−0.024	*PTP4A2*
chr17	751731	753661	17	1.12 × 10^−15^	−0.063	−0.009	*DBIL5P*, *GEMIN4*
chr16	69131682	69134198	18	1.16 × 10^−15^	−0.060	−0.011	*UTP4*, *DERPC*, *CHTF8*
chr3	192514288	192515909	7	1.31 × 10^−15^	−0.067	−0.017	*FGF12-AS2*, *FGF12*
chr1	197774465	197776165	21	1.34 × 10^−15^	−0.058	−0.009	*DENND1B*
chr2	69643394	69645055	15	1.84 × 10^−15^	−0.063	−0.008	*ANXA4*, *AAK1*
chr5	151531039	151532008	5	2.18 × 10^−15^	−0.055	−0.012	*FAT2*
chr2	3604333	3606880	12	2.66 × 10^−15^	0.053	0.009	*COLEC11*
chr8	31032193	31034963	36	2.69 × 10^−15^	−0.060	−0.004	*WRN*, *PURG*
chr9	93095295	93096947	17	2.69 × 10^−15^	−0.108	−0.013	*CARD19*, *AL451065.1*
chr19	35114963	35117967	13	2.69 × 10^−15^	−0.052	−0.015	*AC020907.6*, *FXYD3*
chr3	63863019	63864891	17	2.96 × 10^−15^	−0.055	−0.007	*ATXN7*, *THOC7*

**Table 3:** Gene Ontology enriched for DMPs in all ASD individuals predicted by the Enrichr platform.

Category	Enriched Term	Overlap	*p*-Value	Adjusted *p*-Value	Key Associated Genes
GO Biological Process	Anterograde Trans-Synaptic Signaling (GO:0098916)	21/189	9.05 × 10^−4^	0.891	*SYT5*; *MPP2*; *KCNIP1*; *KCNC4*; *CACNA1B*; *SLC1A2*; *PRG2*; *HTR2B*; *GAD2*; *SLC6A1*; *HTR5A*; *SDCBP*; *CACNB3*; *GRIN2A*; *GABRR1*; *HRH2*; *KCNQ2*; *HRH4*; *PCDHB6*; *CRH*; *DLGAP1*
	Negative Regulation of Lipid Localization (GO:1905953)	4/10	1.20 × 10^−3^	0.891	*NFKBIA*; *CSRP1*; *NR1H2*; *TNF*
	Cellular Response to Epidermal Growth Factor Stimulus (GO:0071364)	6/28	2.79 × 10^−3^	0.891	*DUSP22*; *MBD4*; *GAREM1*; *DAB2IP*; *INPP5K*; *ZPR1*
	Chemical Synaptic Transmission (GO:0007268)	24/260	4.98 × 10^−3^	0.891	*SYT5*; *MPP2*; *KCNIP1*; *KCNC4*; *PRG2*; *CACNA1B*; *SLC1A2*; *HTR2B*; *GAD2*; *LIN7A*; *SLC6A1*; *HTR5A*; *SDCBP*; *GRIN2A*; *GABRR1*; *CACNB3*; *TH*; *HRH2*; *KCNQ2*; *HRH4*; *PCDHB6*; *RIMBP2*; *CRH*; *DLGAP1*
	Positive Regulation of Interleukin-1 Production (GO:0032732)	9/63	5.20 × 10^−3^	0.891	*AIM2*; *IFI16*; *CARD8*; *STAT3*; *NLRP2*; *S100A13*; *USP50*; *TNF*; *TMEM106A*
	Cellular Response to Tumor Necrosis Factor (GO:0071356)	10/87	1.51 × 10^−2^	0.891	*NFKBIA*; *ASAH1*; *AIM2*; *DAB2IP*; *INPP5K*; *CIB1*; *TNF*; *TP53*; *FCAR*; *BIRC2*
	Ras Protein Signal Transduction (GO:0007265)	8/68	2.48 × 10^−2^	0.891	*RASSF1*; *RAPGEF1*; *NF1*; *RRAS2*; *USP50*; *HRAS*; *SOS2*; *TP53*
	MHC Class II Protein Complex Assembly (GO:0002399)	3/13	2.74 × 10^−2^	0.891	*HLA-DMB*; *HLA-DQA2*; *HLA-DPA1*
GO Cellular Component	tRNA Methyltransferase Complex (GO:0043527)	3/10	1.29 × 10^−2^	0.970	*TRMT112*; *TRMT10C*; *TRMT11*
	Azurophil Granule Lumen (GO:0035578)	10/89	1.75 × 10^−2^	0.970	*DNAJC3*; *SDCBP*; *PRDX5*; *CREG1*; *FUCA1*; *DEFA3*; *MAN2B1*; *MPO*; *PRSS2*; *CTSC*
	MHC Protein Complex (GO:0042611)	4/20	1.83 × 10^−2^	0.970	*HLA-DMB*; *HLA-C*; *HLA-DQA2*; *HLA-DPA1*
	MHC Class II Protein Complex (GO:0042613)	3/14	3.35 × 10^−2^	0.970	*HLA-DMB*; *HLA-DQA2*; *HLA-DPA1*
GO Molecular Function	Calcium Ion Binding (GO:0005509)	29/324	3.40 × 10^−3^	0.861	*COLEC11*; *ITSN1*; *TCHH*; *ITLN1*; *CIB1*; *TPGS1*; *SLC8A1*; *LOXL2*; *SCGN*; *CRACR2A*; *CSRP1*; *S100A13*; *CDH24*; *EFHD1*; *S100A14*; *PRSS3*; *PRSS2*; *S100A11*; *S100A10*; *TRPM4*; *SYT5*; *KCNIP1*; *S100A1*; *PLA2G4C*; *HPCAL1*; *RUNX1*; *PLSCR1*; *PCDHB6*; *ESYT3*
	Bubble DNA Binding (GO:0000405)	3/7	4.24 × 10^−3^	0.861	*NEIL3*; *WRN*; *ERCC5*
	I-SMAD Binding (GO:0070411)	4/15	6.34 × 10^−3^	0.861	*SMAD2*; *SMAD4*; *SMAD3*; *SMAD7*
	Neutral L-amino Acid Transmembrane Transporter Activity (GO:0015175)	6/38	1.31 × 10^−2^	0.861	*SLC36A2*; *SLC43A2*; *SLC6A15*; *SLC1A2*; *SLC38A9*; *SLC38A4*
	Voltage-Gated Monoatomic Cation Channel Activity (GO:0022843)	8/75	4.14 × 10^−2^	0.861	*CACNB3*; *KCNE1*; *CATSPER4*; *KCNH8*; *KCNC4*; *KCNQ2*; *CACNA1B*; *TMC2*

**Table 4:** Hypergeometric distribution analysis of DMP-associated genes and SFARI gene categories across ASD severity groups.

Group	SFARI Category	SFARI Genes	Overlap Count	Adjusted *p*-Value
All ASD	All_SFARI	1158	62	0.001
	Score_1	219	15	0.018
	Score_2	663	33	0.027
	Score_3	201	11	0.094
	Score_Unscored	75	3	0.469
	Syndromic	270	15	0.059
	Non-syndromic	888	47	0.005
	1_Syndromic	108	8	0.055
	2_Syndromic	48	0	1.000
	3_Syndromic	39	4	0.063
	1_Non-syndromic	111	7	0.094
	2_Non-syndromic	615	33	0.015
	3_Non-syndromic	162	7	0.340
Moderate ASD	All_SFARI	1158	87	<0.001
	Score_1	219	18	0.018
	Score_2	663	49	0.001
	Score_3	201	14	0.099
	Score_Unscored	75	6	0.129
	Syndromic	270	22	0.010
	Non-syndromic	888	65	<0.001
	1_Syndromic	108	10	0.039
	2_Syndromic	48	2	0.624
	3_Syndromic	39	4	0.129
	1_Non-syndromic	111	8	0.129
	2_Non-syndromic	615	47	0.001
	3_Non-syndromic	162	10	0.182
Severe ASD	All_SFARI	1158	62	0.001
	Score_1	219	15	0.020
	Score_2	663	32	0.028
	Score_3	201	12	0.046
	Score_Unscored	75	3	0.454
	Syndromic	270	16	0.028
	Non-syndromic	888	46	0.005
	1_Syndromic	108	8	0.042
	2_Syndromic	48	1	0.785
	3_Syndromic	39	4	0.052
	1_Non-syndromic	111	7	0.086
	2_Non-syndromic	615	31	0.026
	3_Non-syndromic	162	8	0.179

**Table 5:** Reproducible consensus epigenetic signatures between our cohorts and independent studies.

Reference Study	Probe ID	Gene Symbol	All ASD (logFC)	Mod ASD (logFC)	Sev ASD (logFC)	Ref. Cohort Effect	Direction
Perini et al., 2023 (EPIC Blood) [32]	cg05640346	*CNTNAP2*	−0.68	−0.667	−0.7	−0.017 (logFC)	Hypomethylated
	cg14859916	*CNTNAP2*	−0.596	−0.623	−0.582	−0.024 (logFC)	Hypomethylated
	cg13861180	*GRIN1*	−0.386	−0.385	−0.396	−0.008 (logFC)	Hypomethylated
	cg02224047	*CUX1*	0.46	0.503	0.445	+0.006 (logFC)	Hypermethylated
	cg10482632	*CSRP1*	−0.516	−0.409	−0.53	−0.004 (logFC)	Hypomethylated
Nardone et al., 2014 (BA10 Brain) [31]	cg03409108	*PACSIN1*	−0.308	−0.322	−0.303	−0.064 (Δβ)	Hypomethylated
	cg03301498	*MED29*	−0.31	−0.259	−0.303	−0.074 (Δβ)	Hypomethylated
	cg23071186	*TNFSF14*	−0.584	−0.657	−0.611	−0.087 (Δβ)	Hypomethylated
Nardone et al., 2014 (BA24 Brain) [31]	cg11669284	*WDR70*	−0.394	−0.343	−0.417	−0.125 (Δβ)	Hypomethylated
	cg12157614	*AGAP1*	−0.408	−0.379	−0.407	−0.092 (Δβ)	Hypomethylated
	cg25117092	*MED12L*	−0.585	−0.678	−0.521	−0.052 (Δβ)	Hypomethylated
Siu et al., 2019 (16p11.2del Blood) [33]	cg12154538	*NCOR2*	0.748	0.794	0.665	+0.155 (Δβ)	Hypermethylated
	cg24975642	*NCOR2*	0.667	0.694	0.62	+0.121 (Δβ)	Hypermethylated
Siu et al., 2019 (CHD8 Blood) [33]	cg17192377	*HDHD2*	−0.355	−0.343	−0.355	−0.064 (Δβ)	Hypomethylated
Corley et al., 2019 (Young Brain) [28]	cg18107827	*FAM110D*	−0.321	−0.338	−0.324	−0.116 (Δβ)	Hypomethylated
Kimura et al., 2019 (Blood) [30]	cg04131969	*MYADML*	3.202	3.899	3.172	+0.158 (Δβ)	Hypermethylated
Nardone (BA24) [31] and Perini (EPIC) [32]	cg09384035	*LINC01181*	−0.245	−0.233	−0.252	-	Hypomethylated

## Data Availability

The data supporting the findings of this study are available from the corresponding author upon reasonable request.

## Supplementary Materials

The following supporting information can be downloaded at: https://www.mdpi.com/article/10.3390/ijms27104161/s1.

## Abbreviations

The following abbreviations are used in this manuscript:

ASD	Autism Spectrum Disorder
CARS-2	Childhood Autism Rating Scale, Second Edition
CTRL	Typically Developing Controls
DSM-5	Diagnostic and Statistical Manual of Mental Disorders, Fifth Edition
M-CHAT	Modified Checklist for Autism in Toddlers
PDDSQ	Pervasive Developmental Disorders Screening Questionnaire
WASI-II	Wechsler Abbreviated Scale of Intelligence-Second Edition
WPPSI-IV	Wechsler Preschool and Primary Scale of Intelligence-Fourth Edition
DMP	Differentially Methylated Position
DMR	Differentially Methylated Region
iPSC	Induced Pluripotent Stem Cell
MHC	Major Histocompatibility Complex
PBMC	Peripheral Blood Mononuclear Cell
SFARI	Simons Foundation Autism Research Initiative
TNF	Tumor Necrosis Factor
ChEA	ChIP Enrichment Analysis
EpiDISH	Epigenetic Dissection of Intra-Sample-Heterogeneity
FDR	False Discovery Rate
GEO	Gene Expression Omnibus
GO	Gene Ontology
logFC	Log Fold Change
PCA	Principal Component Analysis
ssNoob	Single-Sample Normal-Exponential Out-of-Band
|Δβ|	Absolute Delta Beta-Value (methylation difference)
