# Supplementary material for: Genome-Wide DNA Methylation Profiling of Peripheral Blood Mononuclear Cells Reveals Epigenetic Signatures in Autism Spectrum Disorder
Source: Int J Mol Sci. 2026 May 7;27(10):4161. doi: 10.3390/ijms27104161 (PMC13207186; doi:10.3390/ijms27104161)
Supplement: Supplementary file 1 [file ijms-27-04161-s001.zip › Figure S1.pdf]

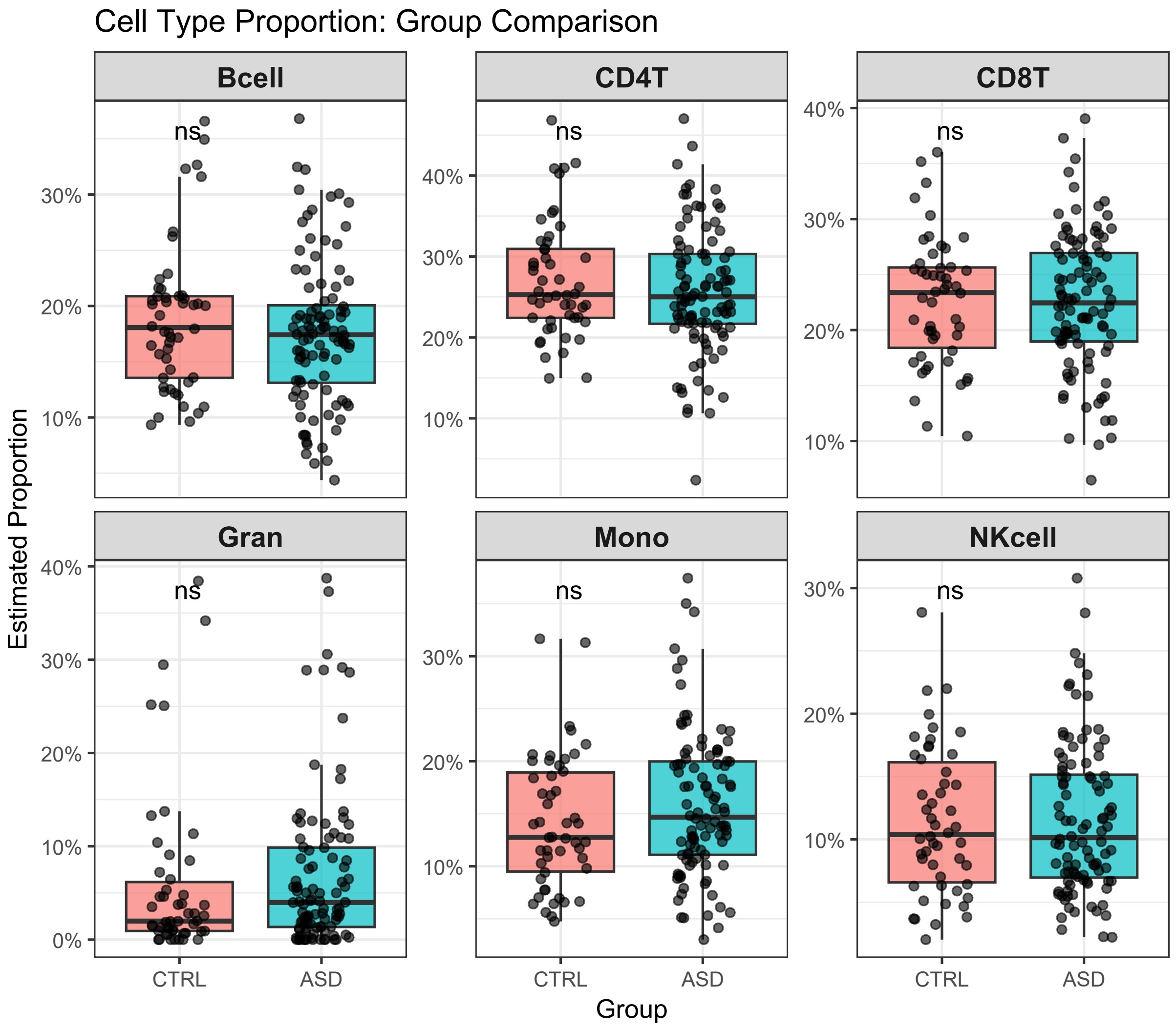

Cell Type	P-value (Mann-Whitney U)	Adjusted P-value	Effect Size (Cohen's d)
Monocytes	0.1407	0.4577	0.2524
Granulocytes	0.1526	0.4577	0.1134
B Cells	0.2721	0.5441	-0.1847
CD8+ T-Cells	0.5808	0.7915	-0.1137
CD4+ T-Cells	0.6596	0.7915	-0.1203
NK Cells	0.992	0.992	0.0188
