# Supplementary material for: Genome-Wide DNA Methylation Profiling of Peripheral Blood Mononuclear Cells Reveals Epigenetic Signatures in Autism Spectrum Disorder
Source: Int J Mol Sci. 2026 May 7;27(10):4161. doi: 10.3390/ijms27104161 (PMC13207186; doi:10.3390/ijms27104161)
Supplement: Supplementary file 1 [file ijms-27-04161-s001.zip › Supplementary Table S1_Demographic information.pdf]

Supplementary Table S1.1: Demographic information of all participants in this study.

Variables	ASD group	TD group
Age (years)		
Mean	6.90	7.17
SD	±4.00	±3.86
Sex		
Number of males (frequency)	83 (83%)	42 (84%)
Number of females (frequency)	17 (17%)	8 (16%)

Supplementary Table S1.2: Demographic information of the participants when stratified ASD based on severity.

Variables	Mild ASD	Mild matched CTRL	Moderate ASD	Moderate matched CTRL	Severe ASD	Severe Matched CTRL
Age (years)						
Mean	7.50	7.50	7.20	7.34	6.61	7.17
SD	±4.60	±4.66	±3.97	±3.78	±3.98	±3.86
Sex						
Number of Males (frequency)	7 (87.5%)	7 (87.5%)	29 (80.6%)	29 (80.6%)	47 (83.9%)	42 (84%)
Number of Females (frequency)	1 (12.5%)	1 (12.5%)	7 (19.4%)	7 (19.4%)	9 (16.1%)	8 (16%)
