# Supplementary figures and images for: Genome-Wide DNA Methylation Profiling of Peripheral Blood Mononuclear Cells Reveals Epigenetic Signatures in Autism Spectrum Disorder
Source: Int J Mol Sci. 2026 May 7;27(10):4161. doi: 10.3390/ijms27104161 (PMC13207186; doi:10.3390/ijms27104161)

**A**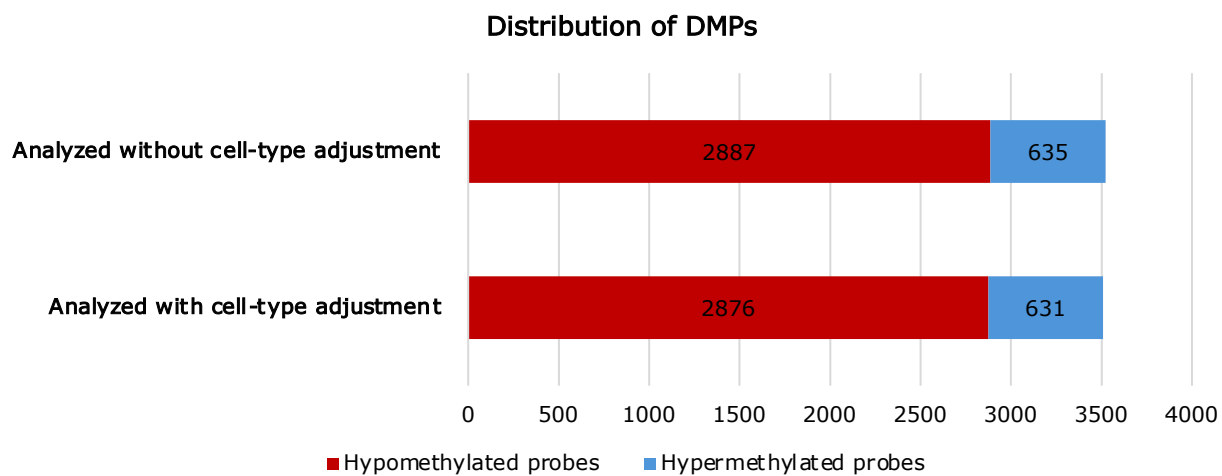**B**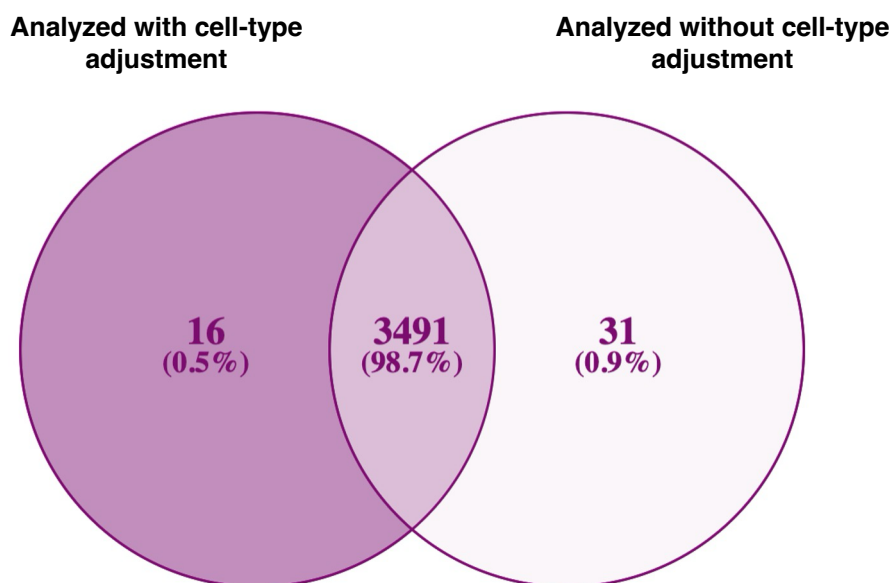

Supplement: Supplementary file 1 [file ijms-27-04161-s001.zip › Figure S2.pdf]
